# Combined analysis of ZAP-70 and CD38 expression in sudanese patients with B-cell chronic lymphocytic leukemia

**DOI:** 10.1186/s13104-019-4319-8

**Published:** 2019-05-23

**Authors:** Ameen Abdulaziz Basabaeen, Enaam Abdelrhman Abdelgader, Othman Saeed BaHashwan, Ebtihal Ahmed Babekir, Nour Mahmoud Abdelateif, Salem Ahmed Bamusa, Saadia Osman Abdelrahim, Osama Ali Altayeb, Eman Abbass Fadul, Ibrahim Khider Ibrahim

**Affiliations:** 1 Department of Hematology, Faculty of Medical Laboratory Sciences, Al Neelain University, Khartoum, Sudan; 2 Department of Pathology, Faculty of Medicine, Al Neelain University, Khartoum, Sudan; 3 Department of Family Medicine, Faculty of Medicine, University of Gezira, Wad Medani, Sudan; 4 Department of Hematology, School of Medical Sciences, University Sains Malaysia, Health Campus, 16150 Kubang Kerian, Kelantan Malaysia; 5 Flow Cytometry Laboratory for Leukemia & Lymphoma Diagnosis, Khartoum, Sudan; 6 Ministry of Health & Population, Hadhramout, Yemen

**Keywords:** B-CLL, ZAP-70, CD38, Combined, Rai, Binet, Hematological, Sudan

## Abstract

**Objective:**

To investigate the ZAP-70 and CD38 expressions and their combined expressions in Sudanese B-CLL patients and their relationships with clinical and hematological characteristics as well as the disease staging at presentation.

**Results:**

In the present cross-sectional descriptive study, analysis of ZAP-70 expression showed that 36/110 (32.7%) patients positively expressed ZAP-70 and insignificant higher presentation in intermediate and at advanced stages as well as no correlation was seen with hematological parameters and clinical features compared with negatively ZAP-70, on the other hand, 41/110 (37.3%) were CD38^+^ and no significant correlation was shown with the stage at presentation, clinical characteristics (except Splenomegaly, P = 0.02) and hematological parameters. However, in combined expressions of both ZAP-70 and CD38 together, 20/110 (18.2%) were concordantly ZAP-70^+^/CD38^+^, 53/110 (48.2%) concordantly ZAP-70^−^/CD38^−^ and 37/110 (33.6%) either ZAP-70^+^ or CD38^+^, and these three groups showed insignificant correlation with clinical (except Splenomegaly, P = 0.03) and hematological parameters, and the stage at presentation. Our data showed the combined analysis of these two markers, lead to classify our patients into three subgroups (either concordant positive, negative or discordant expressions) with statistically insignificant correlation with clinical presentation (except Splenomegaly), hematological parameters and stage at presentation of B-CLL patients.

**Electronic supplementary material:**

The online version of this article (10.1186/s13104-019-4319-8) contains supplementary material, which is available to authorized users.

## Introduction

B-cell chronic lymphocytic leukemia (B-CLL) is characterized by progressive accumulation of monoclonal, small, mature-appearing CD5^+^ B-cells in the peripheral blood, bone marrow and secondary lymphoid tissue [1]. CLL is the most common leukemia in adults in western countries and it is more common in males [2, 3]. B-CLL is a heterogeneous disorder characterized by a variable clinical course [4]. In a continual effort to identify patients with poor prognosis and to facilitate the clinical management of B-CLL [5].

Staging systems have delineated the clinical presentation and natural history of B-CLL and have allowed predicting survival and treatment requirements [6, 7]. However, the staging systems lack the ability to distinguish prospectively patients with early stage B-CLL that will rapidly progress to aggressive disease from patients destined to remain in early stage for a long period of time [4].

The presence or absence of somatic mutations in the (IgVH) of B-CLL cells has been described as one of the most powerful prognostic factors distinguishing two disease subsets [8, 9]. The cases with mutated (IgVH) genes exhibit a favorable clinical course and they may never require treatment [10]; while patients with unmutated (IgVH) genes are characterized by a reduced survival and responsiveness to chemotherapy [8, 11, 12]. However, determination of (IgVH) mutation is based on DNA sequencing which is not always available for routine clinical use. So, several surrogate markers [13], that correlate with (IgVH) mutational status have been identified. Moreover, CD38 and ZAP-70 expressions have been proposed as less expensive and time-consuming surrogates for (IgVH) mutational status [14, 15]. More recently the combined of ZAP-70 and CD38 expressions analysis provided complementary prognostic information to identify three patient subgroups with good, intermediate and poor prognosis [16]. Many studies showed that (CD38^−^/ZAP-70^−^) group of patients with good prognosis, (CD38^+^/ZAP-70^+^) group with poor prognosis, and group (CD38^+^/ZAP-70^−^) or CD38^−^/ZAP-70^+^) with intermediate prognosis [16, 17].

To the best of our knowledge, this is the first study conducted in Sudan to investigate the combined expressions of both ZAP-70 and CD38 in B-CLL patients and their Correlation with clinical features, hematological parameters and at a stage of presentation. The aim of our study to evaluate the effect of ZAP-70 and CD38 independently as well as combined expression of both together in B-CLL patients and their influence in clinical features, hematological parameters and at a stage of presentation, accordingly our patients are divided into three groups.

## Main text

### Methods

This study was a prospective cross-sectional descriptive study, conducted in Khartoum state, Sudan, in the period from April 2017 to April 2018. A total of 110 blood samples were collected from patients with B-cell CLL. Patients were obtained at Flow Cytometry Laboratory for Leukemia & Lymphoma Center, Khartoum, Sudan, where they were referred for immunophenotypic diagnosis.

All patients were diagnosed based on clinical history, physical examination, complete blood count, immunophenotypic criteria and B lymphocytes ≥ 5×10^9^/l, according to IWCLL [17]. The stage of the CLL was assessed by Rai and Binet [6, 7] classification. All patients were newly diagnosed without any previous B-CLL treatment; as explained in our previous work [18].

#### Determination of blood count and immunophenotyping

Samples were collected in EDTA tubes, complete blood counts were performed by using automated hematological analyzer (SYSMEX- KX-21N, Japan).

The diagnosis of CLL was confirmed for each patient by flow cytometry (EPICS XL Beckman Coulter Flow Cytometer, Miami, FL, USA), standard protocol of Beckman Coulter [19] was used in fluorescent dye labeled monoclonal antibody for CD45, CD5, CD3, CD19, CD20, CD22, CD23, FMC7, CD79b, kappa, and lambda light chain. A marker was considered positive at a cutoff level of ≥ 30% according to the BCSH guideline [20]. A diagnostic scoring system was assessed by Matutes et al. [21, 22]. Absolute B lymphocyte count was obtained by flow cytometry. Expression of ZAP-70 and CD38were performed by flow cytometry, as previously described [18, 23, 24].

#### Statistical analysis

Patient’s data was collected by a structural interview questionnaire and from patient’s medical records and analyzed by using the (SPSS), version-23. The analysis was done for quantitative variables of B-cell CLL to compare means and variance of means by using T-test, ANOVA and Kruskal–Wallis and Mann–Whitney test as well as correlations with Pearson and Spearman.

### Results

#### ZAP-70 and CD38 expression

ZAP-70 expression was positive in 36/110 (32.7%) and negative in 74/110 (67.3%) whereas CD38 was positively expressed in 41/110 (37.3%) and negative in 69/110 (62.7%). The correlation of expression of ZAP-70 and CD38 with clinical features, hematological parameter, and stages according to Rai and Binet staging systems are shown in Additional file 1: Tables S1–S3.

#### Combined ZAP-70 and CD38 expression analysis

When ZAP-70 and CD38 were combined expressions together, 20/110 (18.2%) were concordant positive (ZAP-70^+^ CD38^+^), 53/110 (48.2%) were concordant negative (ZAP-70^−^ CD38^−^), and 37/110 (33.6%) were discordant. In discordant groups, positivity of ZAP-70 protein without CD38 expression (ZAP-70^−^ CD38^+^) was detected in 16 patients (14.5%), whereas 21 patients (19.1%) were CD38-positive but ZAP-70-negative (ZAP-70^−^ CD38^+^). The correlation of combined ZAP-70 and CD38 with clinical features, hematological parameters and stages at presentation according to Rai and Binet staging systems are shown in Tables 1, 2 and 3.

**Table 1:** Combined ZAP-70 and CD38 expression within clinical parameters

Parameter(s)	Concordant		Discordant	P* value
	+VE no (%)	−VE no (%)	No (%)	
Gender				
Male	16 (80.0%)	38 (71.7%)	25 (67.6%)	0.71
Female	4 (20.0%)	15 (27.3%)	12 (32.4%)	
Lymphadenopathy	16 (80.0%)	34 (64.2%)	28 (75.7%)	0.30
Splenomegaly	14 (61.1%)	20 (43.2%)	20 (63.4%)	0.03
Hepatomegaly	4 (20.0%)	7 (13.2%)	3 (8.1%)	0.43
No. of lymph areas involved				
≤ 3	12 (60%)	34 (64.2%)	19 (51.4%)	0.55
> 3	8 (40%)	19 (35.8%)	18 (48.6%)	

Except Splenomegaly, there was insignificant correlation between concordant or discordant ZAP-70/CD38 expressions with clinical characteristics

X^2^ test (n = 110), P* value significant below 0.05

**Table 2:** Means of hematological parameters of combined ZAP-70 and CD38 expressions

Parameter(s) Mean ± SD	Concordant		Discordant	P* value
	+VE (%)	−VE (%)	(%)	
TWBCs × 10^3^/μl	93.4 ± 73.5	97.2 ± 76.5	86.4 ± 76.6	0.80
Platelets × 10^3^/μl	207.7 ± 88.7	200.3 ± 118.3	163.4 ± 89.0	0.17
Absolute lymphocytes × 10^3^/μl	80.1 ± 69.0	87.5 ± 73.2	75.6 ± 63.7	0.73
Monoclonal B lymphocytes × 10^3^/μl	72.0 ± 67.6	77.78 ± 67.9	67.5 ± 66.5	0.77
Hemoglobin g/dl	11 ± 3	11 ± 3	11 ± 2	0.43

There was no significant correlation between concordant or discordant ZAP-70/CD38 expressions with hematological parameters

ANOVA test; (n = 110), P* value significant below 0.05

**Table 3:** Combined ZAP-70 and CD38 expressions according modified Rai and Binet stages

Parameter(s)	Concordant		Disconcordant	P* value
	+VE (%)	−VE (%)	(%)	
Rai stage				
Low risk (0)	1 (5%)	6 (11.3%)	3 (8.1%)	0.72
Intermediate (I, II)	10 (50%)	23 (43.4%)	13 (35.1%)	
High risk (III, IV)	9 (45%)	24 (45.3%)	21 (56.8%)	
Binet stage				
A	6 (30%)	19 (35.8%)	8 (21.6%)	0.69
B	7 (35%)	15 (28.4%)	13 (35.2%)	
C	7 (35%)	19 (35.8%)	16 (43.2%)	
Total	20/110 (18.2%)	53/110 (48.2%)	37/110 (33.6%)	

Analysis of combined ZAP-70 and CD38 expressions according modified Rai and Binet stages showed no significant correlation

Kruskal–Wallis test; (n = 110). P* value significant below 0.05

### Discussion

ZAP-70 and CD38 were used as surrogate markers for the mutated and unmutated (IgVH) to facilitate the clinical management of B-CLL.

Considering ZAP-70, using 20% as the cutoff for positivity, 36/110 (32.7%) of our patients had positive ZAP-70 expression. Nearly similar distribution was seen by Del Giudice et al. [25], Hus et al. [26], D’arena et al. [27] (28%, 36.5% and 36% respectively), whereas Waheed et al. [28] reported higher expression (60%) but Gogia et al. [29] and Abdel-Gader et al. [23] reported lower expression (25%) and (22.6%) in India and Sudan, respectively. According to meta-analysis done by Liu et al. [30] the role of ZAP-70 expression in the prognosis of B-CLL is unaffected by region. Discrepancies may be due to different [protocols, the antibody used and the cutoff value was used (10% or 20%)] [27, 31].

In our study, from patients who were ZAP-70 positive, (52.8%) were at high risk (III, IV) Rai stage, (5.6%) were at low risk (0) Rai stage. On the other hand, (35%) were at C Binet stage and (30%) were at A Binet stage at while in ZAP-70^−^ patients (47.3%) were at high risk (III, IV) stage, (10.8%) at low risk (0) stage, (35.8%) at Binet C stage, and (35.8%) at Binet A stage. The lower Percentage of ZAP-70^+^ patient presented at low risk (0) Rai stage compared to ZAP-70^−^ patients (5.6% and 10.8%, respectively) indicated much aggressive disease at presentation in ZAP-70^+^ patient, but was statistically insignificant. While Hus et al. [26], Schroers et al. [16], D’arena et al. [27], Del Giudice et al. [25] and Waheed et al. [28] found that there were significant correlations of ZAP-70 with stage at presentation, our study found no such associations (Additional file 1: Table S3), similar to study that reported by Abdel-Gader et al. [23], and Gogia et al. [29].

Regarding CD38 expression, using 30% cutoff for positivity, 41/110(37.3%) patients were positive, nearly same as that reported by Abdelgader et al. [24] and Gogia et al. [29], (36.4% and 36.25%) respectively, whereas Waheed et al. [28] reported higher expression (60%), but D’arena et al. [27] reported lower expression (29%). Expression variations may be due to different [protocols, an antibody used and variation of cutoff used for positivity (7%, 20% and 30%)] [25, 27]. Nearly same distribution trends in Rai and Binet stages as in ZAP-70 were noticed for CD38 (Additional file 1: Table S3).

Hus et al. [26], Schroers et al. [16], D’arena et al. [27], Del Giudice et al. [25] and Waheed et al. [28] found that there were significant correlations of CD38 with the stage at presentation but our study found no such associations (Additional file 1: Table S3), same as the study that reported by Abdelgader et al. [24], and Gogia et al. [29], but noteworthy, Abdelgader et al. [24] only found that there was significant association between CD38 and hemoglobin concentration by using cutoff level 7% [24] and Schroers et al. [16]. Hus et al. [26] however, found there were significant differences in hematological parameter with expressions of ZAP-70 and CD38. In our study, the only exception was Splenomegaly (P = 0.021) with CD38 expression and no significant correlations of ZAP-70 and CD38 expressions with age, sex, hematological parameters and clinical findings at presentation were found (Additional file 1: Table S1–S3), which was inconsistent with Abdelgader et al. [24], Gogia et al. [29], Waheed et al. [28].

The correlation of ZAP-70 with CD38 showed a strong association (P = 0.003) (Additional file 2: Figure S1), which agree with the results of the literature. Accordingly, our patients were categorized into three subgroups, two concordant (ZAP-70^+^ CD38^+^), (ZAP-70^−^ CD38^−^), and discordant group where only ZAP-70 or CD38 is positive. Our separate analysis of both discordant groups (ZAP-70^−^ CD38^+^) and (ZAP-70^+^ CD38^−^) revealed that there were no clinical or biological differences so they were treated as one discordant group.

When comparing between (ZAP-70^+^ CD38^+^) and (ZAP-70^−^ CD38^−^), Hus et al. [26], Schroers et al. [16], D’arena et al. [27] found that there was significant correlation of (ZAP-70^+^ CD38^+^) with clinical presentation and hematological parameters while Gogia et al. [29] failed to find such associations in 80 B-CLL patients of whom 56% were concordant (ZAP-70^+^ CD38^+^). The correlation with discordant groups was much less significant through previous studies. Furthermore, Hus et al. [26], Schroers et al. [26], D’arena et al. [27] found that there was a significant correlation between (ZAP-70^+^ CD38^+^) with the survival rates and treatment free interval. This association was not evaluated in this study.

In our study, of 20/110 concordant (ZAP-70^+^ CD38^+^) patients, 19/20 (95%) were at intermediate (I, II) or advanced (III, IV) Rai stage comparing to only 1/20 (5%) in early stage (0), and in 37/110 concordant (ZAP-70^−^ CD38^−^) patients 34/37 (81.9%) were at intermediate (I, II) or high risk (III, IV) Rai stage. Our results showed no significant difference between concordant (ZAP-70^+^ CD38^+^) and (ZAP-70^−^ CD38^−^) with Rai or Binet stage at presentation and the correlation with discordant groups was much less significant (Table 3); the same conclusion was reported by Gogia et al. [29], Assem et al. [32]. The majority of our patients 100/110 (91%) were presented at the intermediate or advanced stage; this can explain the comparable correlation between the two concordant groups and with discordant groups.

With the exception of Splenomegaly (P = 0.037), regarding age, sex, clinical findings, and hematological parameters means, there were no significant correlations between concordant ZAP-70 and CD38 expressions or with discordant expression of both (Tables 1, 2 and 3), same as Gogia et al. [29], Waheed et al. [28] reported, and in contrast to Hus et al. [26], Del Giudice et al. [25] who found that there were significant differences in the most hematological parameter between concordant groups.

Noticeably, Splenomegaly was correlated with positive expression of CD38 but not ZAP-70, and the combination of both (ZAP-70^+^ CD38^+^) was still significant which may imply the significance of concordant analysis of ZAP-70 and CD38 as a better prognostic marker better than each one independently. Frequencies of concordant and discordant expressions of ZAP-70 and CD38 in some important studies are shown in Additional file 1: Table S4.

Conclusion, our study showed CD38^+^ was significantly associated with Splenomegaly. While expressions of ZAP-70 and CD38 were not statistically associated with significantly more advanced stages at Rai and Binet of B-CLL patients at presentation. In addition, the combination of these two markers together leads to classifying our patients into three subgroups (concordant positive, negative and discordant groups) which is statistically insignificant with clinical presentation (except Splenomegaly), hematological parameters and stage at presentation of B-CLL patients.

## Limitations

Limitations are worth to mention such as sampling method which depends on voluntary participation, patients were not followed up for progression, survival rates and response to treatment after diagnosis confirmation. Finally, our study does not include studying the prognostic value of ZAP-70 or CD38 independently or even combined the expression of both together, indeed all limitations that are mentioned above should be taken in consideration in order to the interpretation of our results.

## Additional files

**Additional file 1: Table S1.** Frequencies of clinical parameters according to ZAP-70 and CD38 expressions. **Table S2.** Means of hematological parameters according to ZAP-70 and CD38 expressions. **Table S3.** ZAP-70 and CD38 expressions in modified Rai and Binet stages. **Table S4.** Combined ZAP-70 and CD38 expressions in some previous studies and present study.

**Additional file 2: Figure S1.** Relationship between ZAP-70 and CD38 expressions in 110 B-CLL patients.

## Data Availability

The individual data are available in the archives of the Flow Cytometry for Leukemia & Lymphoma Diagnosis, Khartoum, Sudan and can be obtained from the corresponding author on request.

## Abbreviations

CLL: chronic lymphocytic leukemia
IWCLL: International workshop of chronic lymphocytic leukemia
CD: cluster of differentiation
IgHV: immunoglobulin heavy chain variable region
ZAP-70: zeta chain associated protein
EDTA: ethylenediamine tetraacetic acid
SPSS: Statistical Package for Social Sciences
WBC: white blood cell
RBC: red blood cell
BCSH: British Committee for Standards in Hematology
