# Supplementary material for: Combined analysis of ZAP-70 and CD38 expression in sudanese patients with B-cell chronic lymphocytic leukemia
Source: BMC Res Notes. 2019 May 23;12:282. doi: 10.1186/s13104-019-4319-8 (PMC6533771; doi:10.1186/s13104-019-4319-8)
Supplement: Supplementary file 1 — Additional file 1: Table S1. Frequencies of clinical parameters according to ZAP-70 and CD38 expressions. Table S2. Means of hematological parameters according to ZAP-70 and CD38 expressions. Table S3. ZAP-70 and CD38 expressions in modified Rai and Binet stages. Table S4. Combined ZAP-70 and CD38 expressions in some previous studies and present study. [file 13104_2019_4319_MOESM1_ESM.docx]

**Table S1:** Frequencies of clinical parameters according to ZAP-70 and CD38 expressions.

| **Parameter** | | | | **ZAP-70** | | **P* value** | **CD38** | | **P* value** |
| --- | --- | --- | --- | --- | --- | --- | --- | --- | --- |
|  |  |  |  | **+VE (%)**  **N=36** | **-VE (%)**  **N=74** |  | **+VE (%)**  **N=41** | **-VE (%)**  **N=69** |  |
| **Age** | | **< 63 years** | | **15(41.67%)** | **33(44.6%)** | **0.77** | **17(41.46%)** | **31(44.93%)** | **0.72** |
|  |  | **≥ 63 years** | | **21(58.33%)** | **41(55.4%)** |  | **24(58.54%)** | **38(55.07%)** |  |
| **Gender** | **Male**  **Female** | | | **26(72.2%)** | **53(71.6%)** | **0.95** | **31(75.6%)** | **48(69.6%)** | **0.5** |
|  |  |  |  | **10(27.8%)** | **21(28.4%)** |  | **10(24.4%)** | **21(30.4%)** |  |
| **Lymphadenopathy** | | | | **27(75.0%)** | **51(68.9%)** | **0.51** | **33(80.5%)** | **45(65.2%)** | **0.09** |
| **Splenomegaly** | | | | **22(61.1%)** | **32(43.2%)** | **0.08** | **26(63.4%)** | **28(40.6%)** | **0.021** |
| **Hepatomegaly** | | | | **5(13.9%)** | **9(12.2%)** | **0.8** | **6(14.6%)** | **8(11.6%)** | **0.65** |
| **No. of Lymph areas involved** | | | **≤ 3** | **18(50%)** | **27(36.5%)** | **0.427** | **25(61%)** | **40(58%)** | **0.49** |
|  |  |  | **>3** | **18(50%)** | **47(63.5%)** |  | **16(39%)** | **29(42%)** |  |

**Mann-Whitney Test. (n=110) *P** value significant below 0.05**

There were no significant correlations between ZAP-70 and CD38 expressions with clinical characteristics, the only exception splenomegaly in CD38.

**Table S2:** Means of Hematological parameters according to ZAP-70 and CD38 expressions.

| **Parameter** | **ZAP-70** | | **P* value** | **CD38** | | **P* value** |
| --- | --- | --- | --- | --- | --- | --- |
|  | **+VE (%)** | **-VE (%)** |  | **+VE (%)** | **-VE (%)** |  |
| TWBCs×10^3^/ul | **92.7±67.6** | **92.9±79.4** | **0.98** | **87.7±80.2** | **95.9±72.9** | **0.58** |
| Platelets×10^3^/ul | **186.4±89.1** | **190.6±112.4** | **0.84** | **186.4±92.7** | **190.9±112.2** | **0.82** |
| Absolute Lymphocytes×10^3^/ul | **80.6±63.7** | **82.9±74.6** | **0.87** | **73.6±73.3** | **86.1±69.7** | **0.45** |
| Monoclonal B-Lymphocytes×10^3^/ul | **73.2±61.9** | **73.3±69.6** | **0.99** | **66.9±70.5** | **77.1±64.9** | **0.44** |
| Hemoglobin g/dl | **11.2±2.5** | **11.2±2.4** | **0.98** | **11.1±2.6** | **11.2±2.4** | **0.75** |

**Independent T test; (n=110) *P** value significant below 0.05**

No significant correlation has been seen for ZAP-70 and CD38 expressions with hematological parameters.

**Table S3:** ZAP-70 and CD38 expressions in Modified Rai and Binet stages.

| **Parameter** | **ZAP-70** | |  | | |  | **CD38** | | | |
| --- | --- | --- | --- | --- | --- | --- | --- | --- | --- | --- |
|  | **+VE (%)** | **-VE (%)** | | **P* value** | **+VE (%)** | | | **-VE (%)** | **P* value** |  |
| **Rai stage** |  |  | |  |  | | |  |  |  |
| **Low risk(0)** | **2(5.6%)** | **8(10.8%)** | | **0.64** | **3 (7.3%)** | | | **7 (10.1%)** | **0.86** |  |
| **Intermediate(I,II)** | **15(41.7%)** | **31(41.9%** | |  | **18 (43.9%)** | | | **28 (40.6%)** |  |  |
| **High risk (III,IV)** | **19(52.8%)** | **35(47.3%)** | |  | **20 (48.8%)** | | | **34 (49.3%)** |  |  |
| **Binet stage** |  |  | |  |  | | |  |  |  |
| **A** | **9 (25.0%)** | **24 (32.4%)** | | **0.51** | **11 (26.8%)** | | | **22 (31.9%)** | **0.81** |  |
| **B** | **14 (38.9%)** | **21 (28.4%)** | |  | **13 (31.7%)** | | | **22 (31.9%)** |  |  |
| **C** | **13 (36.1%)** | **29 (39.2%)** | |  | **17 (41.5%)** | | | **25 (36.2%)** |  |  |
| **Total** | **36/110 (32.7%)** | **74/110 (67.3%)** | |  | **41/110 (37.3%)** | | | **69/110 (62.7%)** |  |  |

**Kruskal-Wallis Test (n=110).*P** value significant below 0.05**

No significant correlation has been noticed for ZAP-70 and CD38 expressions with stage at presentation.

**Table S4:** Combined ZAP-70 and CD38 expressions in some previous studies and present study.

| **Study** | **Concordant +ve** | **Concordant –ve** | **Disconcordant** |
| --- | --- | --- | --- |
| **Del Giudice et al., 2005[25]** | 46/197(23%) | 70/197 (36%) | 81/197 (41%) |
| **Schroers et al., 2005[16]** | 59/252(23.4%) | 120/252 (47.6%) | 73/252 (29%) |
| **Hus et al., 2006[26]** | 35/118(22.4%) | 83/118(53.2%) | 38/118(24.4%) |
| **Assem et al., 2007[32]** | 5/50(10%) | 24/50(48%) | 21/50(42%) |
| **D’arena et al., 2007[27]** | 27/157(17%) | 81/157 (52%) | 49/157 (31%) |
| **Gogia et al., 2013[29]** | 11/80(13.75%) | 34/80(42.5%) | 35/80(43.75%) |
| **WAHEED et al., 2015[28]** | 17/30(56.6%) | 11/30(36.7%) | 2/30(6.7%) |
| **Present study** | 20/110 (18.2%) | 53/110 (48.2%) | 37/110 (33.6%) |

This table shows the variable frequencies of combined ZAP-70 and CD38 expressions in some important studies. The range of concordant positivity from 10 to 56.6%

When comparison with other subgroups is made, many studies confirmed a strong association of the concordant ZAP-70+ CD38+ with B-CLL disease aggressive progression, decreased overall survival rates, lower treatment free intervals, and decreased responding to treatment Del Giudice et al., 2005, Schroers et al., 2005, D’arena et al., 2007[16, 25, 27], and most of studies showed that there is no association with stages at presentation Assem et al., 2007, Gogia et al., 2013, Hus et al., 2006, WAHEED et al., 2015[26, 28, 29, 32].
