# Supplementary material for: Combined analysis of ZAP-70 and CD38 expression in sudanese patients with B-cell chronic lymphocytic leukemia
Source: BMC Res Notes. 2019 May 23;12:282. doi: 10.1186/s13104-019-4319-8 (PMC6533771; doi:10.1186/s13104-019-4319-8)
Supplement: Supplementary file 2 — Additional file 2: Figure S1. Relationship between ZAP-70 and CD38 expressions in 110 B-CLL patients. [file 13104_2019_4319_MOESM2_ESM.docx]

Figure S1: Relationship between ZAP-70 and CD38 expressions in 110 B-CLL patients.


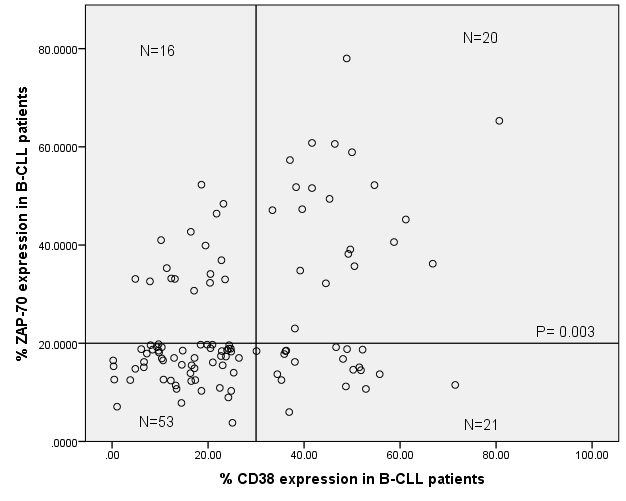


The correlation of ZAP-70 with CD38 showed strongly association (P value =0.003)
